# Therapeutic effects of Isaria felina on postmenopausal osteoporosis: modulation of gut microbiota, metabolites, and immune responses

**DOI:** 10.3389/fimmu.2025.1508634

**Published:** 2025-04-09

**Authors:** Xiaoyan Li, Chenhui Xue, Yongming Yang, Lili Zhao, Lixia Chen, Jing Wang, Lei Yan, Zan Meng, Xiaochen Qiao, Sujiao Liang, Xihua Yang

**Affiliations:** ^1^ Shanxi Province Cancer Hospital/Shanxi Hospital Affiliated to Cancer Hospital, Chinese Academy of Medical Sciences/Cancer Hospital Affiliated to Shanxi Medical University, Taiyuan, Shanxi, China; ^2^ Third Hospital of Shanxi Medical University, Shanxi Bethune Hospital, Shanxi Academy of Medical Sciences, Tongji Shanxi Hospital, Taiyuan, Shanxi, China; ^3^ Department of Orthopedics, Hospital of Shaanxi Provincial Armed Police Corps, Xi’an, Shaanxi, China; ^4^ Department of Orthopedics, The Second Hospital of Shanxi Medical University, Taiyuan, Shanxi, China

**Keywords:** *Isaria felina*, postmenopausal osteoporosis, gut microbiota, 16s rDNA sequencing, metabolomics

## Abstract

**Background:**

The intricate relationship between human health and gut microecology has emerged as a central theme in contemporary medical research. Postmenopausal osteoporosis, primarily driven by estrogen deficiency, remains a major health concern. Traditional Chinese herbal medicines have attracted significant interest for their promising role in osteoporosis treatment.

**Methods:**

The effects of *Isaria felina*, derived from *Cordyceps sinensis*, on postmenopausal osteoporosis in rats are the focus of this study. Adult female Sprague-Dawley rats were categorized into control, postmenopausal osteoporosis (OVX), and *Isaria felina*-treated (IF+OVX) groups. Following a 12-week treatment period, various analyses, including micro-CT, histological assessments, 16S rDNA sequencing, untargeted metabolomics, flow cytometry, and ELISA, were performed.

**Results:**

Micro-CT and histological assessments indicated significant improvements in bone loss and obesity control in OVX rats treated with *Isaria felina*. 16S rDNA sequencing revealed that *Isaria felina* corrected gut microbiota dysbiosis, particularly in the *Bacteroides* and *Ruminococcus genera*. Untargeted metabolomics highlighted alterations in nucleotide and lipid metabolism. Flow cytometry and ELISA analyses demonstrated that *Isaria felina* modulated the Th17/Treg immune balance, resulting in reduced levels of inflammatory cytokines IL-17 and TNF-α.

**Conclusions:**

These findings indicate that *Isaria felina* mitigates bone loss in postmenopausal osteoporosis through modulation of gut microbiota and immune responses, underscoring its potential as a therapeutic agent for osteoporosis treatment.

## 1 Introduction

Osteoporosis is a systemic and metabolic bone disease (BMD) characterized by decreased bone mineral density, disruption of bone tissue microstructure, and increased risk of fractures (1). There are numerous causes of osteoporosis, with common ones including decreased estrogen levels, disuse, glucocorticoid use, and obesity (2–5). Due to the rising prevalence of osteoporosis, osteoporotic fractures are also on a rapid increase, particularly fractures of the spine and hips, which significantly impact patients’ quality of life, increase the risk of complications and mortality, and pose tremendous harm. Osteoporotic fractures have become one of the main factors leading to disability and mortality in the elderly, imposing a heavy burden on families and society (6). Therefore, osteoporosis has increasingly become a pressing global health issue that needs to be addressed. Clinically, drugs that stimulate osteogenic activity, reduce osteoclast activity, or both can be used to treat osteoporosis. However, such drugs have issues with safety and tolerability in long-term treatment, as prolonged use can lead to complications such as osteonecrosis of the jaw (ONJ), gastritis, and atypical fractures. Many patients often discontinue such anti-osteoporosis treatments due to concerns about drug side effects (7). Hence, it is imperative to develop innovative treatment approaches for osteoporosis that offer minimal side effects, high therapeutic effectiveness, and promote sustained patient compliance.

Recent advancements in gut microecology research have highlighted the increasingly significant interaction between human health and gut microecology in both basic and clinical medical research. The human gut microecosystem, characterized by its complexity and diversity, relies on the interdependence and mutual regulation of various gut microbiota to maintain microecological balance and support bodily homeostasis^1^. However, external factors can alter the gut microbiota structure, disrupting this balance and leading to diseases^2^. Consequently, gut microbiota is closely linked to the development and progression of numerous human diseases (8–12). In orthopedics, studies have reported associations between gut microbiota and conditions such as osteoporosis, osteoarthritis, rheumatoid arthritis, and bone tumors (13). Our previous research has also identified the significant role of gut microbiota in osteoporosis development (3, 14). Therefore, gut microbiota presents a promising target for osteoporosis prevention and treatment through the modulation of gut microecology.

The gut microbiota is a collection of microorganisms colonized in the gastrointestinal tract, consisting of approximately 10 trillion bacteria. It represents the largest ecosystem in the human body, with a gut microbiota genome containing roughly 150 times more genes than the human genome (15). The gut microbiota plays pivotal roles in various biological processes of organisms, such as regulating the uptake of nutrients, influencing host growth and energy metabolism, and participating in the modulation of inflammatory responses (16). Increasing research has indicated a close relationship between the homeostasis of the gut microbiota and the occurrence and progression of osteoporosis. Through 16S rDNA sequencing of fecal microbiota, Sun discovered that patients with osteoporosis exhibited notable disparities in their microbial composition, with Klebsiella, Escherichia-Shigella, and Akkermansia serving as biomarkers indicative of OP, whereas Faecalibacterium was predominantly present in the healthy control group (17). Wen constructed a mouse model of postmenopausal osteoporosis and, through fecal microbiota and metabolite analysis, discovered significant alterations in the fecal microbial composition of ovariectomized mice compared to the normal group, with notable differences in bile acids metabolism (18).

Current research on enhancing bone metabolism by regulating gut microbiota primarily focuses on probiotics, prebiotics, and traditional Chinese herbal treatments (19). Given the toxic side effects of existing clinical drugs, extracting active compounds from traditional Chinese herbal medicines and natural products has emerged as a new frontier in osteoporosis treatment. Recent studies have confirmed that traditional Chinese herbal medicines can exert anti-osteoporotic effects by modulating the structure of gut microbiota and its metabolites (20–23). *Cordyceps sinensis*, a valuable traditional Chinese herbal medicine, contains various chemical constituents such as nucleosides, amino acids, and glycosides, which have multiple therapeutic effects, including proven anti-osteoporotic properties. Oral administration of *Cordyceps sinensis* can mitigate osteoporosis in rats subjected to ovariectomy (OVX) and counteract disuse-induced bone loss, as well as preserve the trabecular microarchitecture from deterioration (24). Due to the scarcity and high cost of *Cordyceps sinensis*, researchers have focused on the mycelium from strains isolated and fermented from Cordyceps sinensis. *Isaria felina* (IF), a new strain isolated from *Cordyceps sinensis* and preserved by the China General Microbiological Culture Collection Center (CGMCC), has been recognized for its immunomodulatory functions. However, its anti-osteoporotic effects have not been reported in the literature (25). Excessive immune activation is a primary pathogenic factor in postmenopausal osteoporosis caused by estrogen deficiency. Gut microbiota plays a crucial role in bone immunity by regulating the T-helper 17 (Th17)/regulatory T (Treg) cell balance through the immune system, thereby influencing osteoblast and osteoclast function and affecting bone metabolism (26).

Thus, it is hypothesized that *Isaria felina* may exhibit anti-osteoporotic effects by modifying the structure of gut microbiota and its metabolites. This modification could subsequently regulate the immune system and affect bone metabolism.

## 2 Materials and methods

### 2.1 Animals

**Figure 1:**
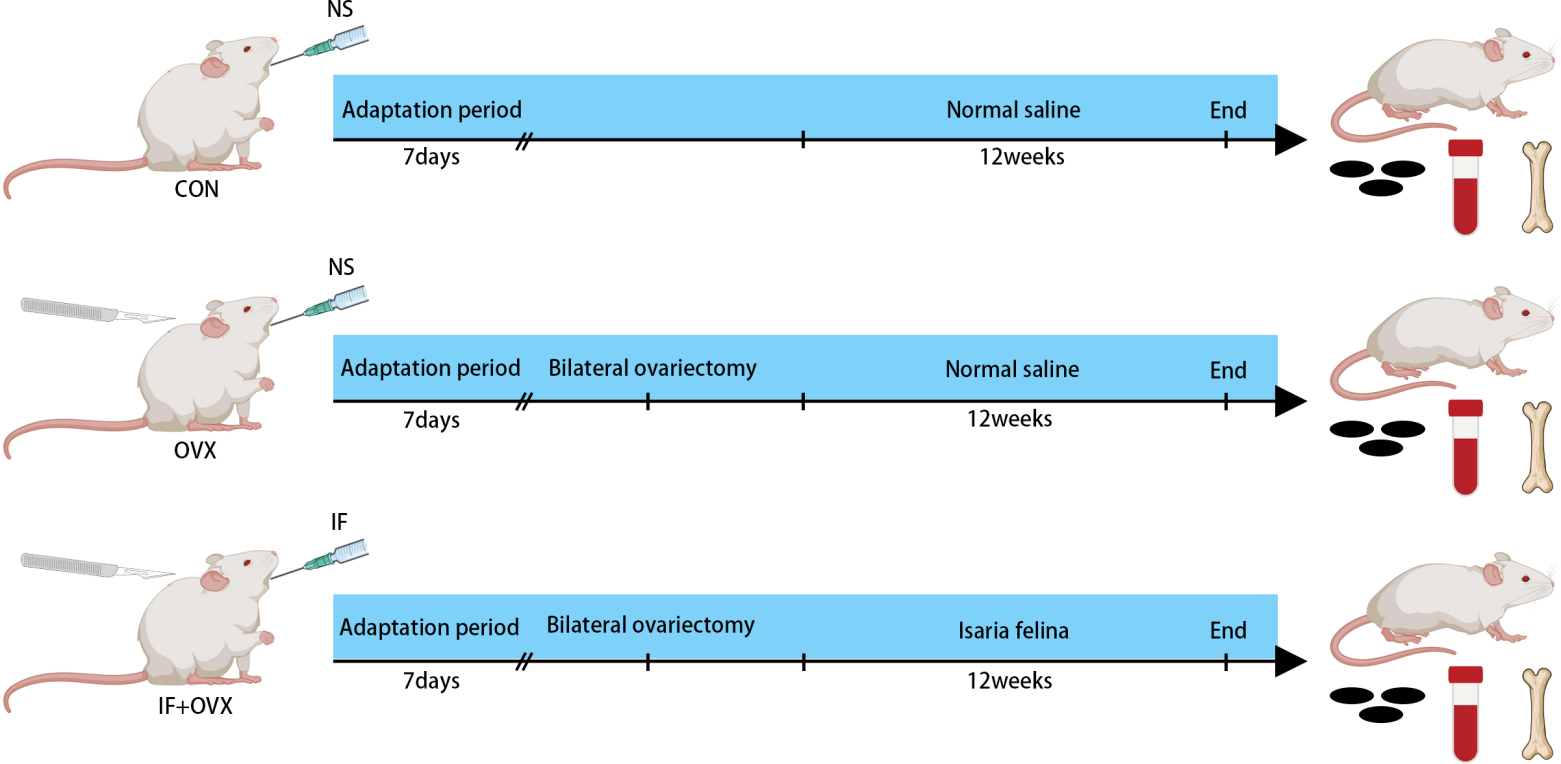
Experimental group allocation and workflow: 21 rats were allocated into three distinct groups (CON, OVX, and IF+OVX).

Adult female Sprague–Dawley rats, weighing 220-250 g, were obtained from the Laboratory Animal Center of Shanxi Cancer Institute (animal production certificate # SCXK (Jin) 2017-0001, Shanxi, China). The rats were housed in a controlled microbial environment at a constant temperature of 23 ± 2°C, with a 12-hour light/dark cycle, and had ad libitum access to sterile food and autoclaved water. After a week of acclimation, the rats were randomly assigned to the control group (CON, 7 rats), postmenopausal osteoporosis group (OVX, 7 rats), and *Isaria felina* treatment group (IF+OVX, 7 rats), with seven rats per group. The OVX and IF+OVX groups underwent bilateral ovariectomy to induce postmenopausal osteoporosis. Following the successful establishment of the model, the rats continued under the same conditions for 12 weeks. During this period, the IF+OVX group received 100 mg/kg of *Isaria felina* daily by oral gavage, while the control group received an equal volume of saline. The 100 mg/kg dose for *Isaria felina* was selected based on our previous study (27). After 12 weeks, the rats were euthanized by CO2 inhalation, and samples were collected for further analysis (**Figure 1**). All animal experiments adhered to the National Institutes of Health (NIH) Guidelines for the Care and Use of Experimental Animals and were approved by the Ethical Committee of Experimental Animal Care of Shanxi Medical University (permit number: 2021014).

### 2.2 Micro-CT scanning

High-resolution micro-computed tomography (MicroCT) (vivaCT80, Scanco, Switzerland) scans of distal femurs were performed to analyze differences in trabecular bone volumes and structures between groups. One hundred contiguous cross-sectional slices above the femoral growth plate were manually selected for analysis. Histomorphometric parameters were computed using Scanco Medical software, determining bone mineral density (BMD), bone volume per tissue volume (BV/TV), trabecular number (Tb.N), trabecular separation (Tb.Sp), cortical bone thickness (Ct.Th), and cortical bone volume (Ct.V) for each sample.

### 2.3 Histological analysis

Femoral samples were fixed in 4% paraformaldehyde (Solarbio, China) for 24 hours and decalcified in 20% ethylenediaminetetraacetic acid (EDTA) solution (Solarbio, China) at 37°C for 6–7 weeks until softened. The femurs were then dehydrated, embedded in paraffin, sectioned into 5-mm longitudinal slices, dried, and stained with hematoxylin and eosin (H&E) and tartrate-resistant acid phosphatase (TRAP) staining kits (Beyotime, China). Morphological characteristics were examined using light microscopy.

### 2.4 Three-point bending test

A three-point bending test was conducted to assess the mechanical properties of the femurs at the middiaphysis using an electronic universal testing machine (ElectroForce 3200 Series, TA Instruments, USA). The femoral samples were positioned and secured on a test stent with two fixed loading points spaced 20 mm apart. A constant displacement rate of 3 mm/min was applied until fractures occurred. Measurements of the internal and external major and minor axis lengths at the fracture points were taken. Peak load and maximum displacement values were recorded.

### 2.5 Flow cytometry

Blood samples were collected from the abdominal aorta of rats in each group. Three milliliters of peripheral blood were drawn into EDTA anticoagulant tubes (BD Vacutainer, USA) and mixed thoroughly. Subsequently, 30 µl of whole blood was transferred to a flow cytometry tube. For T-helper 17 (Th17) cell detection, CD4 (Mouse-Anti-Rat, BD, USA) and interleukin-17 (IL-17) (Thermo Scientific, USA) antibodies were sequentially added. For regulatory T (Treg) cell detection, CD4 (Mouse-Anti-Rat, BD, USA) and CD25 (Mouse-Anti-Rat, BD, USA) antibodies (4.5 µl each) were sequentially added, mixed thoroughly, and incubated for 15 minutes. Next, 200 µl of red blood cell (RBC) lysis buffer (BD Biosciences) was added, mixed thoroughly, and incubated for 10 minutes. The mixture was washed with saline, mixed thoroughly, and centrifuged at 1000 r/min for 5 minutes. The supernatant was discarded, and 400 µl of saline was added, mixed thoroughly, and then analyzed. Flow cytometry determined the proportions of IL-17+CD4+ T cells (Th17) and CD4+CD25+ T cells (Treg).

### 2.6 ELISA

Blood samples were collected from the abdominal aorta of rats and allowed to stand at room temperature for approximately 30 minutes. Following this, the samples were centrifuged at 4°C and 3000 rpm for 20 minutes to separate the serum, which was then stored at -80°C. Interleukin-17 (IL-17), tumor necrosis factor-alpha (TNF-α), interleukin-4 (IL-4), and transforming growth factor-beta (TGF-β) levels were measured using enzyme-linked immunosorbent assay (ELISA) kits (IL-17, TNF-α, IL-4, and TGF-β kits from RayBiotech, USA).

### 2.7 Feces collection

Fecal samples were directly collected from the rats in both groups. At least two fecal pellets were obtained from each rat: one for microbial analysis and the other for metabolic analysis. The samples were placed in sterile centrifuge tubes, immediately frozen in liquid nitrogen, and stored at -80°C for further sequencing.

### 2.8 16S rDNA sequencing

16S rDNA sequencing was performed at Lc-Bio Technologies Co., Ltd. Genomic DNA from fecal samples was extracted using the E.Z.N.A. Stool DNA Kit (Omega Bio-tek, Inc., USA) according to the manufacturer’s instructions. The V3-V4 region of the bacterial 16S rRNA gene was amplified using universal primers 338F (5’-ACTCCTACGGGAGGCAGCAG-3’) and 806R (5’-GGACTACHVGGGTWTCTAAT-3’). The amplified target bands were verified by 1% agarose gel electrophoresis at 170V for 30 minutes. PCR products were purified using the Agencourt AMPure XP nucleic acid purification kit (Beckman Coulter, Inc., USA). The library fragment size was verified using the 2100 Bioanalyzer (Agilent Technologies, Inc., USA). Finally, the library was sequenced on the Illumina Novaseq 6000 platform (Illumina, Inc., USA) with a PE250 sequencing strategy. The 16S rDNA sequencing methodology followed established protocols (17).

### 2.9 16S rDNA microbial community analysis

Sequencing data were split into different samples based on barcode sequences. The data were filtered and merged using Pear (v0.9.6) software. Sequences containing ambiguous bases (N) and those with a quality score below 20 were removed. During merging, the minimum overlap was set to 10 bp, with a p-value of 0.0001. Post-merging, sequences shorter than 230 bp were removed using Vsearch (v2.7.1) software. Chimeric sequences were identified and removed by comparison with the Gold Database using the uchime method. High-quality sequences were clustered into Operational Taxonomic Units (OTUs) using the uparse algorithm in Vsearch (v2.7.1) software, with a sequence similarity threshold of 97%. Alpha diversity and beta diversity analyses were performed using QIIME (v1.8.0) software based on the OTUs and their abundance. The OTU representative sequences were compared with the Silva138 database using the BLAST algorithm, with an e-value threshold of 1e-5, to obtain species classification information for each OTU. Based on the OTU and abundance data analysis, an in-depth exploration of the differences between comparison groups was conducted. To further optimize the data mining process and obtain more valuable information, various advanced analysis methods were employed. This analysis approach was informed by recent methodological advancements (17).

### 2.10 Extraction and UHPLC-MS/MS analysis of fecal metabolites

Initially, 25 mg of samples were mixed with 500 μL of extract solution (methanol: acetonitrile: water = 2:2:1) in an EP tube. The samples were homogenized at 35 Hz for 4 minutes and sonicated in an ice-water bath for 5 minutes, repeated three times. The mixture was centrifuged at 12,000 rpm for 15 minutes at 4°C, and the supernatant was transferred to a fresh glass vial for LC/MS analysis. A QC sample was prepared by combining equal aliquots of the supernatants from all samples. UHPLC-MS/MS analyses were conducted using a UHPLC (Vanquish, Thermo Fisher Scientific, USA) coupled to an Orbitrap Exploris 120 mass spectrometer (Thermo Fisher Scientific, USA) by Beijing Allwegene Technology Co., Ltd. Raw data were converted to mzXML format using ProteoWizard and processed with an in-house program based on XCMS, developed using R, for peak detection, extraction, alignment, and integration. The R package and AllwegeneDB were used for metabolite identification. The methodology used for UHPLC-MS/MS analysis aligns with previously validated techniques (28).

### 2.11 Bioinformatics of fecal metabolome data

Metabolite features detected in more than 50% of experimental samples were excluded from data analysis. Missing values were imputed with half of the minimum value. Internal standard normalization was applied. Features with RSD greater than 30% were omitted from further analysis. The resulting three-dimensional data (peak number, sample name, and normalized peak area) were analyzed using MetaboAnalystR for orthogonal partial least squares discriminant analysis (OPLS-DA). OPLS-DA facilitated better group separation and identification of variables responsible for classification, with R2 and Q2 values indicating explained variation and prediction accuracy, respectively. To validate the robustness and predictive ability of the OPLS-DA model, 200 permutations were performed. The first principal component of variable importance in the projection (VIP) was obtained to refine the analysis. VIP values highlight each variable’s contribution to the model. Metabolites with VIP > 1, P < 0.05 (Student’s t-test), and fold change > 1.5 or < 0.67 were considered significantly altered. Metabolite pathways were identified using the KEGG (http://www.kegg.jp) and MetaboAnalyst (http://www.metaboanalyst.ca/) databases. This bioinformatics workflow follows best practices in metabolomics research (28).

### 2.12 Statistical analysis

All collected data were thoroughly processed using GraphPad Prism 9.0 statistical software. Student’s t-test was employed for statistical analysis between two groups. Differences in gut microbiota between two groups were identified using the Wilcoxon rank-sum test and the Kruskal-Wallis test. Differences in metabolites between groups were determined through univariate analysis, fold-change, and Student’s t-test. A P-value of less than 0.05 was considered statistically significant.

## 3 Result

### 3.1 *Isaria felina* mitigates bone loss in postmenopausal osteoporotic rats

**Figure 2:**
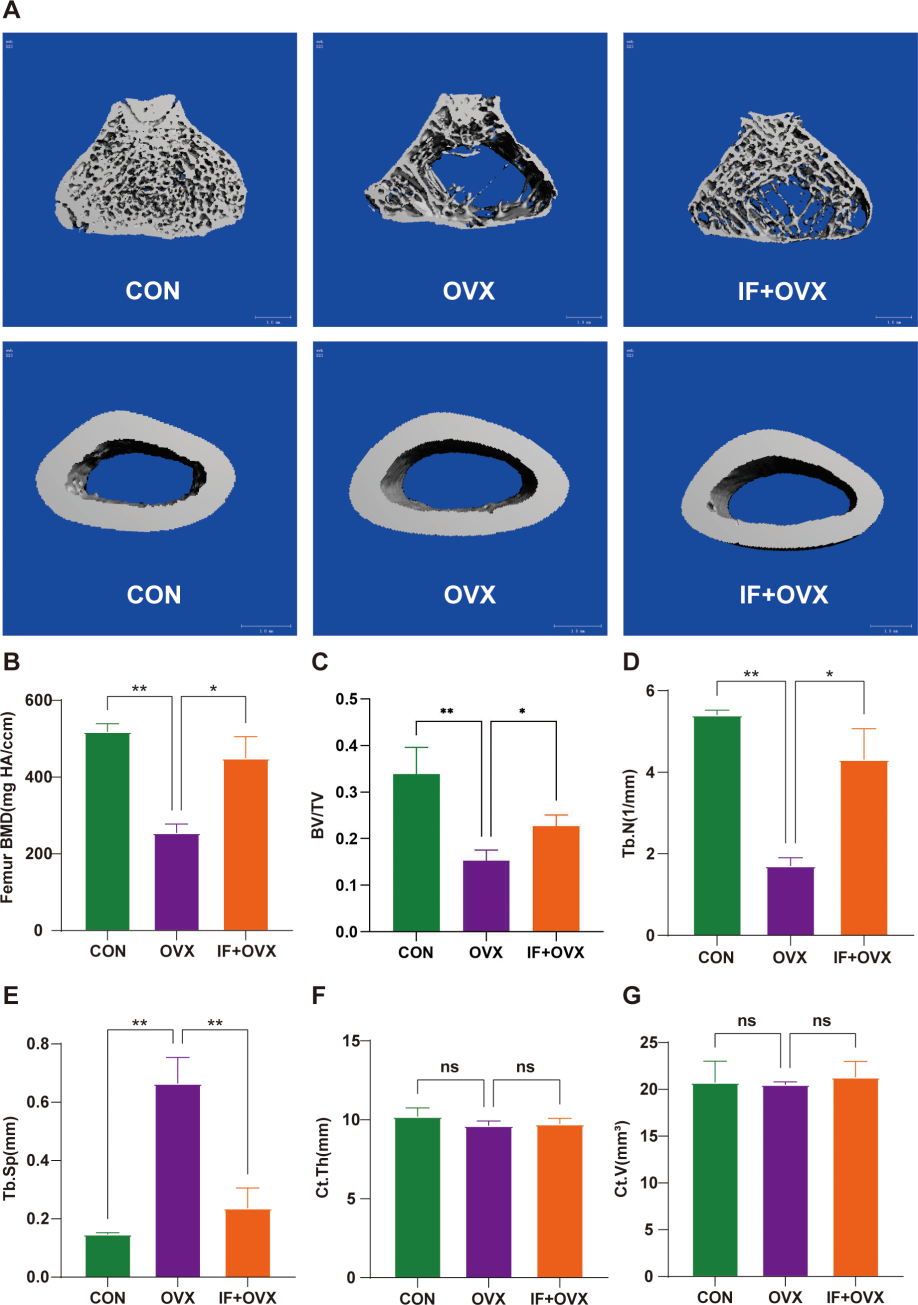
**(A)** Micro-CT 3D reconstruction images of trabecular and cortical bone of the femurs in each group of rats. **(B-G)** Parameters of trabecular and cortical bone in the femurs of each group, including BMD, BV/TV, Tb.N, Tb.Sp, Ct.Th, and Ct.V. Data are presented as mean ± SEM. n = 7, *P < 0.05, **P < 0.01. ns, no significance.

Micro-computed tomography (Micro-CT) analysis was conducted on the femurs of rats in each group. The results demonstrated that oral administration of *Isaria felina* mitigated bone loss in postmenopausal osteoporotic rats. **Figure 2A** presents the Micro-CT 3D reconstruction images of trabecular and cortical bone for each group. Various micro-CT parameters of trabecular and cortical bone, including bone mineral density (BMD), bone volume/total volume (BV/TV), trabecular number (Tb.N), trabecular separation (Tb.Sp), cortical thickness (Ct.Th), and cortical volume (Ct.V), are shown in **Figures 2B–G**. The findings indicated that oral *Isaria felina* significantly improved trabecular bone indices in ovariectomized (OVX) rats but did not significantly affect cortical bone.

**Figure 3:**
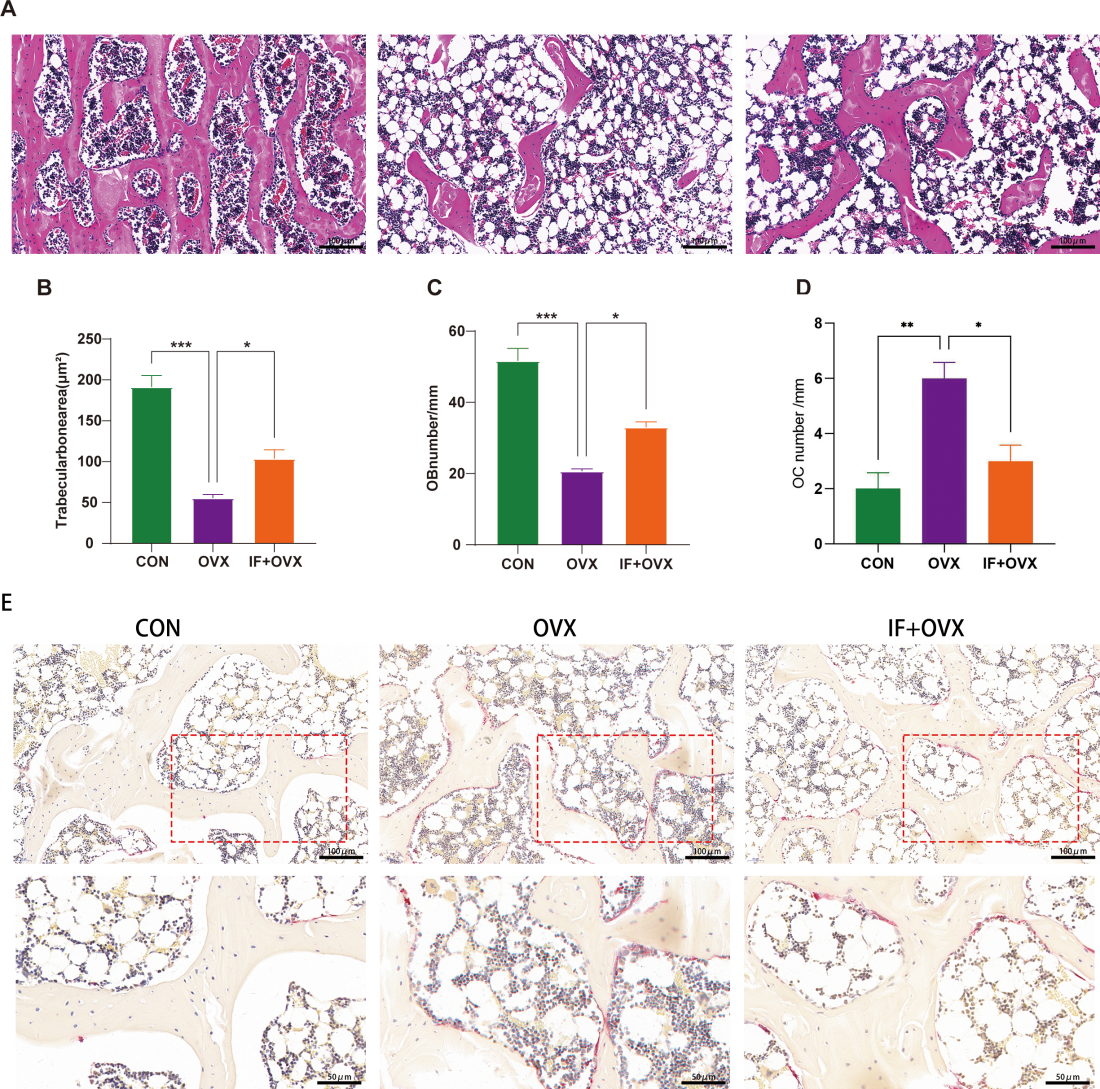
**(A)** HE staining (20x magnification) of the distal femoral metaphysis bone tissue in each group of rats. **(B)** Trabecular bone area ratio. **(C)** Number of osteoblasts. **(D)** Number of osteoclasts. **(E)** TRAP staining (20x, 40x magnification) of the distal femoral metaphysis bone tissue in each group of rats. Data are presented as mean ± SEM. n = 7, *P < 0.05, **P < 0.01, ***P < 0.001.

Histopathological examinations of the femur, including hematoxylin and eosin (HE) staining and tartrate-resistant acid phosphatase (TRAP) staining, were performed to further understand the effects of oral *Isaria felina* on bone morphology in OVX rats. Compared to the control (CON) group, OVX rats exhibited trabecular bone fractures and thinning (**Figure 3A**), a significantly reduced trabecular area ratio, and decreased osteoblast numbers (**Figures 3B, C**). These conditions improved in the IF+OVX group. Additionally, the trabecular bone surface of OVX rats had numerous osteoclasts, indicating significant bone resorption, while the IF+OVX group showed a reduction in osteoclasts on the trabecular surface, resembling the CON group (**Figures 3D, E**). Oral *Isaria felina* effectively inhibited osteoclast activity and improved bone loss caused by postmenopausal osteoporosis.

**Figure 4:**
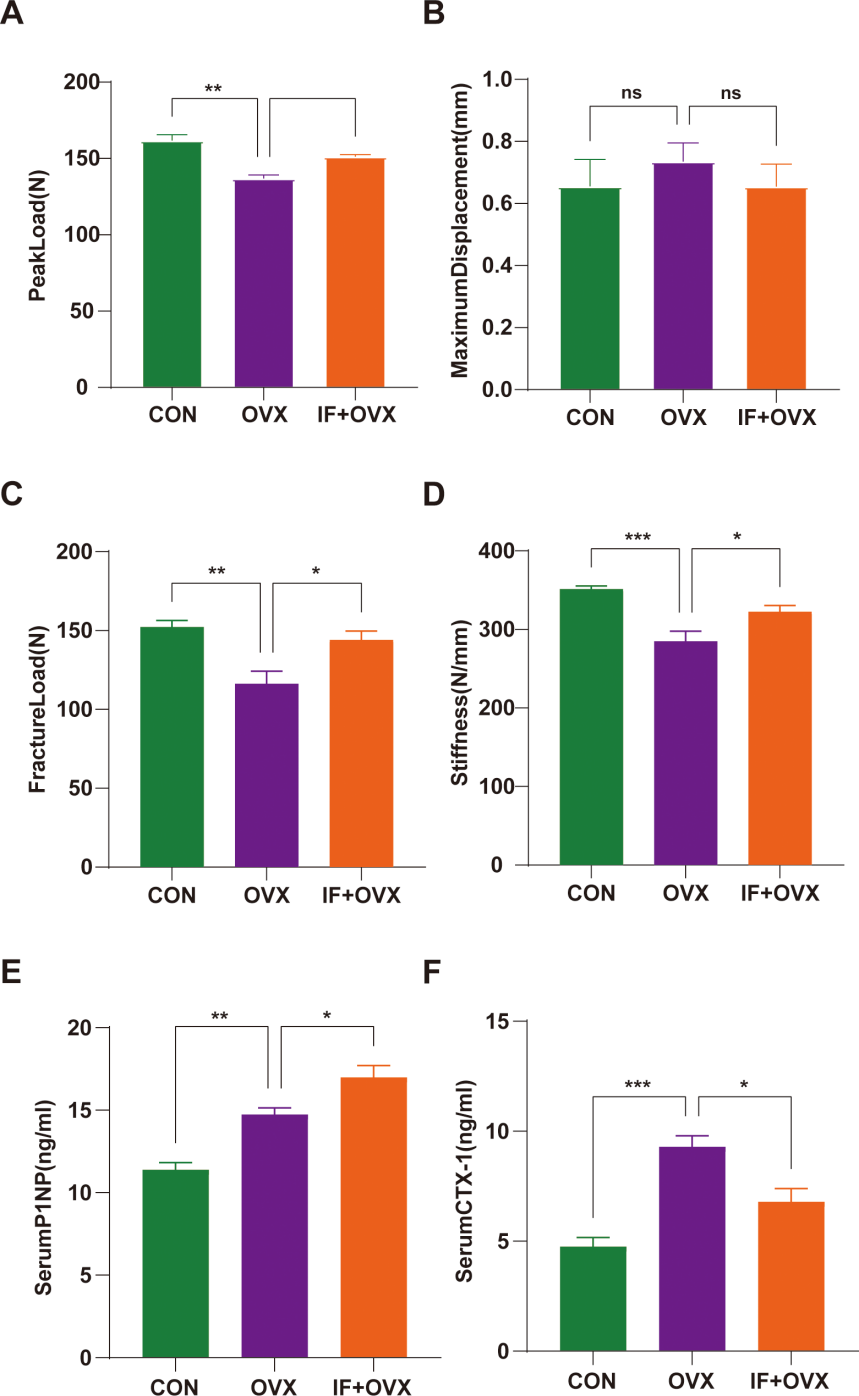
Comparison of three-point bending test parameters between groups. **(A)** Peak load. **(B)** Maximum displacement. **(C)** Fracture Load. **(D)** Stiffness. **(E, F)** Serum levels of bone turnover biomarkers including PINP and CTX-I. Data are presented as mean ± SEM. n = 7, *P < 0.05, **P < 0.01, ns represents no significance.

Stress tests using a three-point bending test were conducted on the femurs of each group of rats. The maximum load-bearing capacity, fracture load, and stiffness of the femurs in the OVX group was significantly reduced compared to the CON group. In the IF+OVX group, the maximum load-bearing capacity, fracture load, and stiffness of the femurs improved, approaching that of the CON group. There was no significant difference in ultimate deflection between the groups (**Figures 4A–D**). The bone resorption and formation markers include CTX-I and PINP, respectively. The ELISA showed that the serum PINP was relatively higher in the OVX model because high bone turnover is associated with postmenopausal OP. The IF+OVX group showed significantly higher serum PINP levels than the OVX group, suggesting improved bone formation with *Isaria felina* treatment (**Figure 4E**). In contrast, serum CTX-I levels (**Figure 4F**) were significantly higher in the OVX group than in the CON group, indicating increased bone resorption. The IF+OVX group exhibited significantly lower serum CTX-I levels compared to the OVX group, indicating a reduction in bone resorption following *Isaria felina* treatment.

### 3.2 *Isaria felina* effectively reduces obesity caused by postmenopausal osteoporosis

Throughout the oral administration of *Isaria felina*, the body weight of each rat group was consistently monitored. Compared to the CON group, the average body weight of OVX rats was notably higher, while the IF+OVX rats showed significantly lower body weight than the OVX rats (**Supplementary Figure S1A**). This suggests that *Isaria felina* effectively alleviates obesity resulting from postmenopausal osteoporosis, potentially contributing to mitigating bone loss. Following the treatment, uterine weight was assessed in each rat group. Both the OVX and IF+OVX groups exhibited markedly reduced uterine weight compared to the CON group (**Supplementary Figure S1B**), confirming the successful establishment of the OVX rat model.

### 3.3 *Isaria felina* alleviates gut microbiota dysbiosis in postmenopausal osteoporotic rats

**Figure 5:**
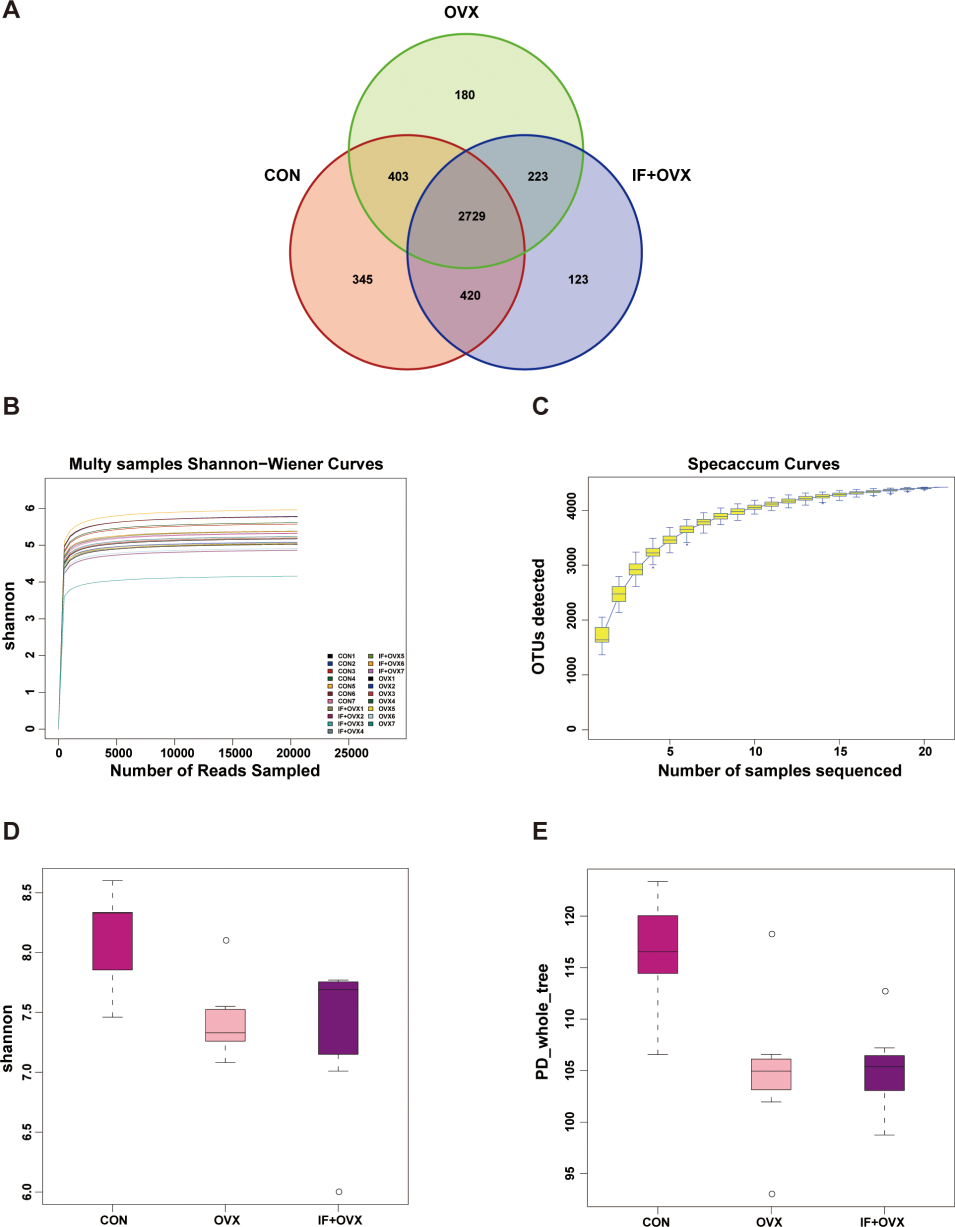
**(A)** Venn diagram showing OTU distribution. **(B)** Rarefaction curve under Shannon index. **(C)** Specaccum species accumulation curve. **(D)** Shannon index. **(E)** PD_whole_tree index. Data are presented as mean ± SEM. n = 7.

A Venn diagram visually represented the shared and unique operational taxonomic units (OTUs) detected in each group through 16S rDNA technology, illustrating the OTU distribution across environmental samples and the intersections among samples or groups. The analysis revealed a total of 4423 OTUs across the three groups: 2729 OTUs were shared among all groups, 345 OTUs were exclusive to the CON group, 180 OTUs were unique to the OVX group, and 123 OTUs were distinctive to the IF+OVX group (**Figure 5A**).

Alpha diversity analysis, based on OTU clustering outcomes, was conducted. The rarefaction curve, generated by randomly selecting individuals from the samples and plotting them against species richness, served to evaluate diversity within samples at different sequencing depths and assess data collection adequacy. A leveling off of the curve indicated sufficient sequencing depth, where increasing data volume yields only a marginal increase in OTUs. Conversely, a continuing rise suggested potential for uncovering additional unknown OTUs with further sequencing, indicating the reasonableness of our sequencing depth (**Figure 5B**).

The species accumulation (Specaccum) curve, a graphical representation of species accumulation with increased sampling effort, was employed to evaluate species composition and estimate diversity within biological communities. This tool confirmed the adequacy of the sample size and estimated potential species richness in biodiversity and community structure investigations (**Figure 5C**).

In studies of microbial diversity, examining diversity within a single sample is pivotal for understanding the richness and diversity characteristics of microbial populations. Using Shannon and phylogenetic diversity (PD)_whole_tree indices, a significant reduction in gut microbial community diversity was observed in OVX rats compared to the CON group, while oral *Isaria felina* supplementation increased community diversity (**Figures 5D, E**).

**Figure 6:**
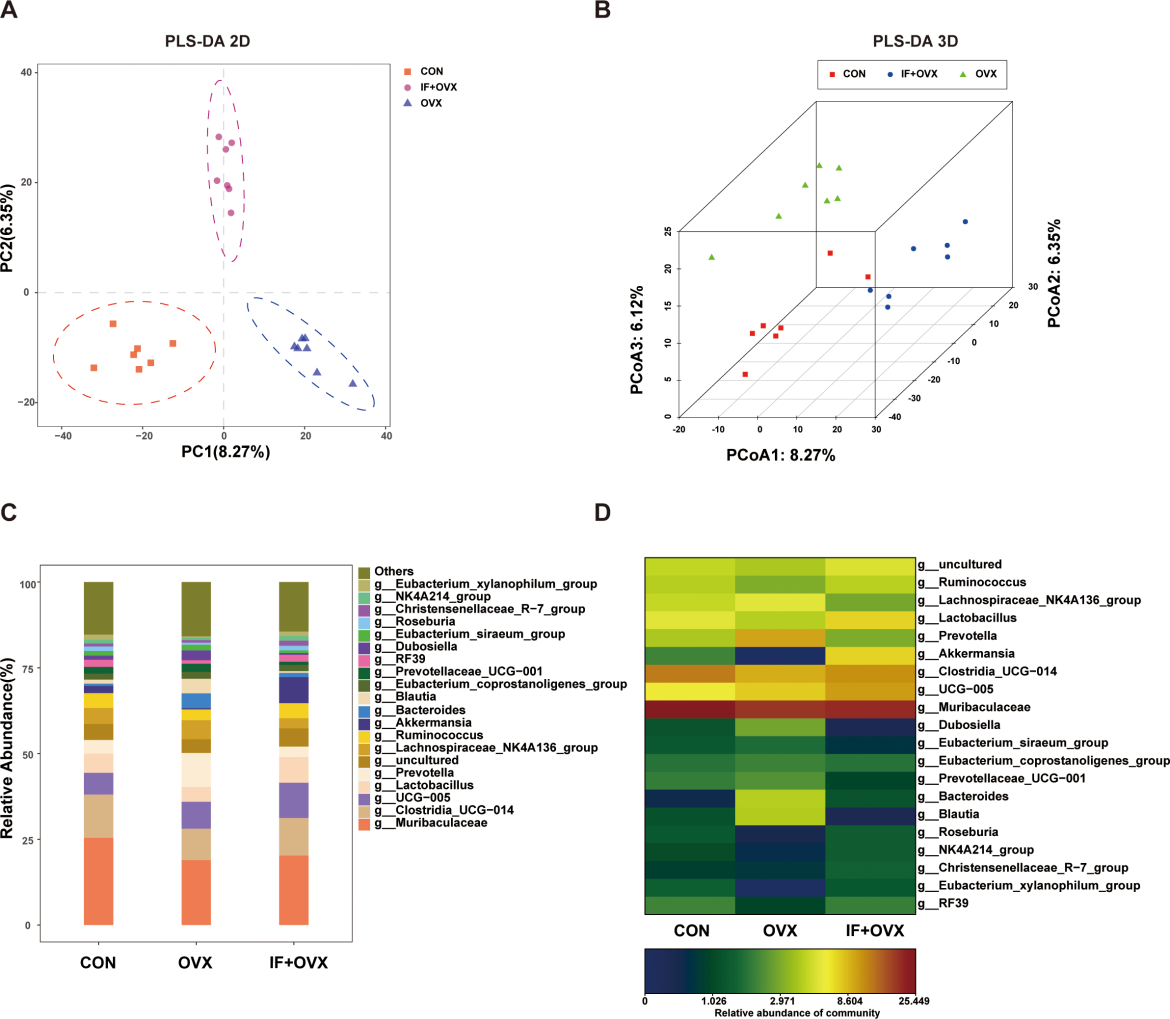
**(A)** 2D PLS-DA of the three groups. **(B)** 3D PLS-DA of the three groups. **(C)** Bar chart showing gut microbiota differences at the genus level among the three groups. **(D)** Heatmap showing gut microbiota differences at the genus level among the three groups. Data are presented as mean ± SEM. n = 7.

Following OTU clustering, beta diversity analysis and species composition assessment were performed. Partial least squares discriminant analysis (PLS-DA) analysis, a supervised discriminant method, unveiled notable discrepancies among categories. The 2D and 3D PLS-DA analyses depicted distinct distributions of microbial communities across each group, highlighting significant differences in gut microbiota composition and abundance among the CON, OVX, and IF+OVX groups (**Figures 6A, B**). Subsequent species composition analysis spanned phylum, class, order, family, genus, and species levels. Genus-level species analysis findings, depicted through bar charts and heatmaps, revealed noteworthy alterations in gut microbiota abundance across various taxonomic levels among the groups (**Figures 6C, D**).

**Figure 7:**
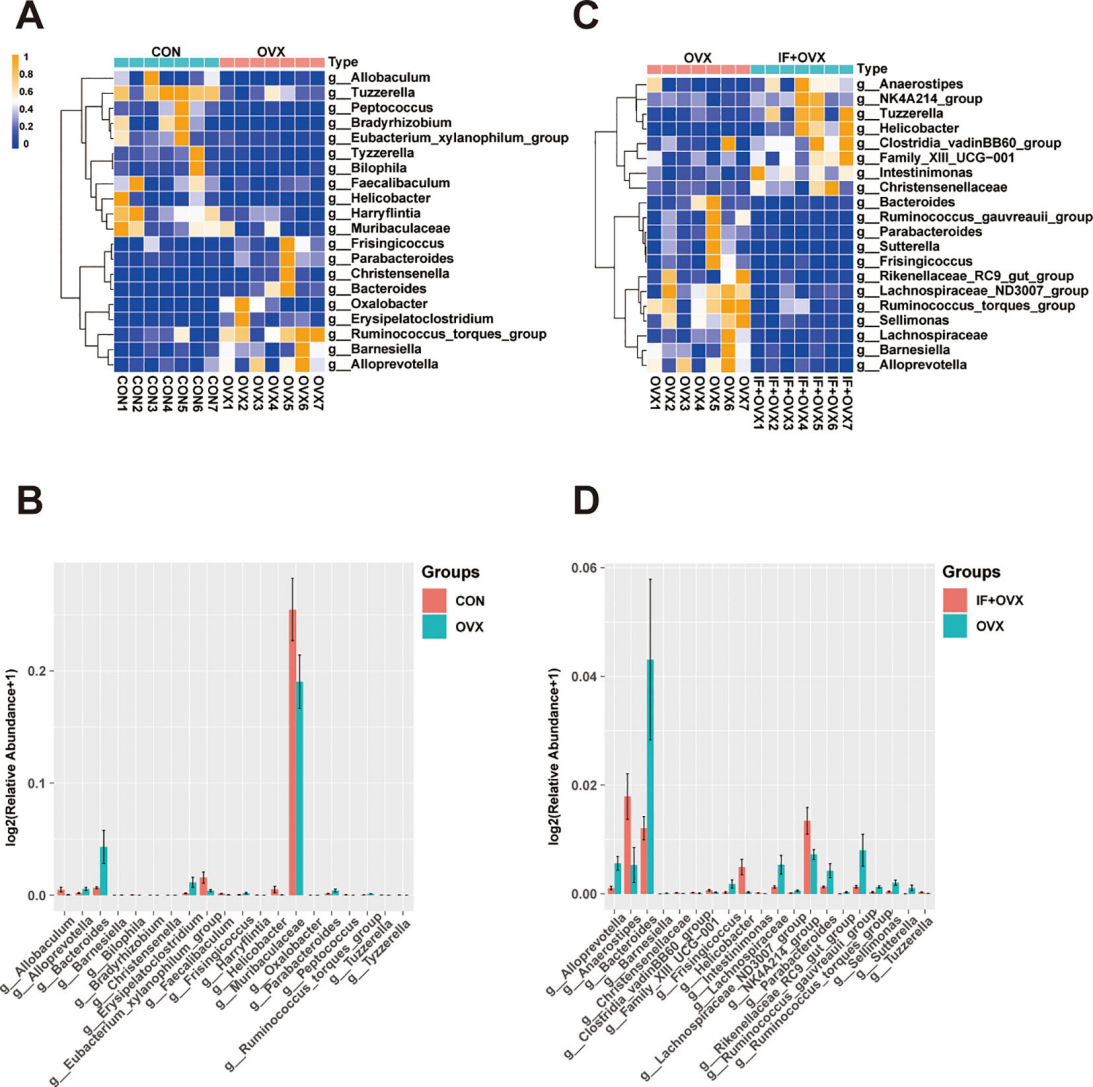
**(A)** Heatmap showing differential species at the genus level between the CON and OVX groups. **(B)** Bar chart showing differential species at the genus level between the CON and OVX groups. **(C)** Heatmap showing differential species at the genus level between the OVX and IF+OVX groups. **(D)** Bar chart showing differential species at the genus level between the OVX and IF+OVX groups. Data are presented as mean ± SEM. n = 7.

A comparative analysis of gut microbiota among the groups showcased significant differences at the genus level through bar charts and heatmaps (**Figures 7A–D**). Specifically, at the genus level, 20 distinct species differentiating the CON and OVX groups, along with 21 distinguishing species between the OVX and IF+OVX groups, were identified. Notably, oral *Isaria felina* supplementation reversed alterations in these species, including genera such as *Bacteroides*, *Tuzzerella*, *Ruminococcus_torques_group*, *Barnesiella*, *Parabacteroides*, *Helicobacter*, *Alloprevotella*, and *Frisingicoccus*.

### 3.4 *Isaria felina* modulates fecal metabolite profiles in postmenopausal osteoporotic rats

**Figure 8:**
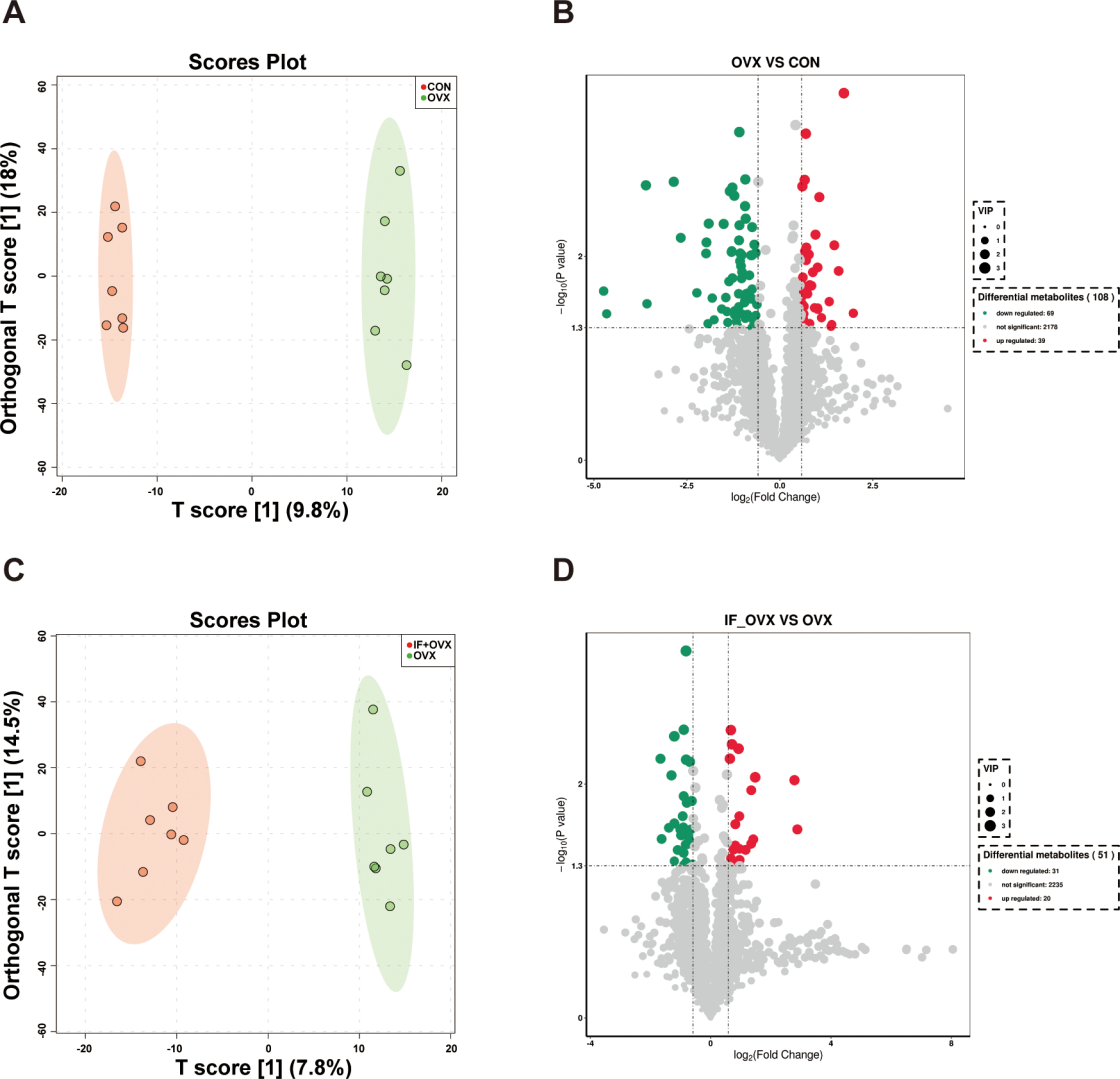
**(A)** OPLS-DA analysis between the CON and OVX groups. **(B)** Volcano plot showing differential metabolites between the CON and OVX groups. **(C)** OPLS-DA analysis between the OVX and IF+OVX groups. **(D)** Volcano plot showing differential metabolites between the OVX and IF+OVX groups. Data are presented as mean ± SEM. n = 7.

Metabolites act as intermediaries through which gut microbiota influences the host. Untargeted metabolomics analysis was conducted on fecal samples from the three rat groups. Orthogonal partial least squares discriminant analysis (OPLS-DA) analysis revealed notable distinctions in fecal metabolite profiles between the CON and OVX groups, as well as between the OVX and IF+OVX groups (**Figures 8A, C**). In this investigation, 108 distinct metabolites were identified between the CON and OVX groups, with 69 downregulated and 39 upregulated in the OVX group (**Figure 8B**). Between the OVX and IF+OVX groups, 51 differential metabolites were identified, with 31 downregulated and 20 upregulated in the IF+OVX group (**Figure 8D**).

**Figure 9:**
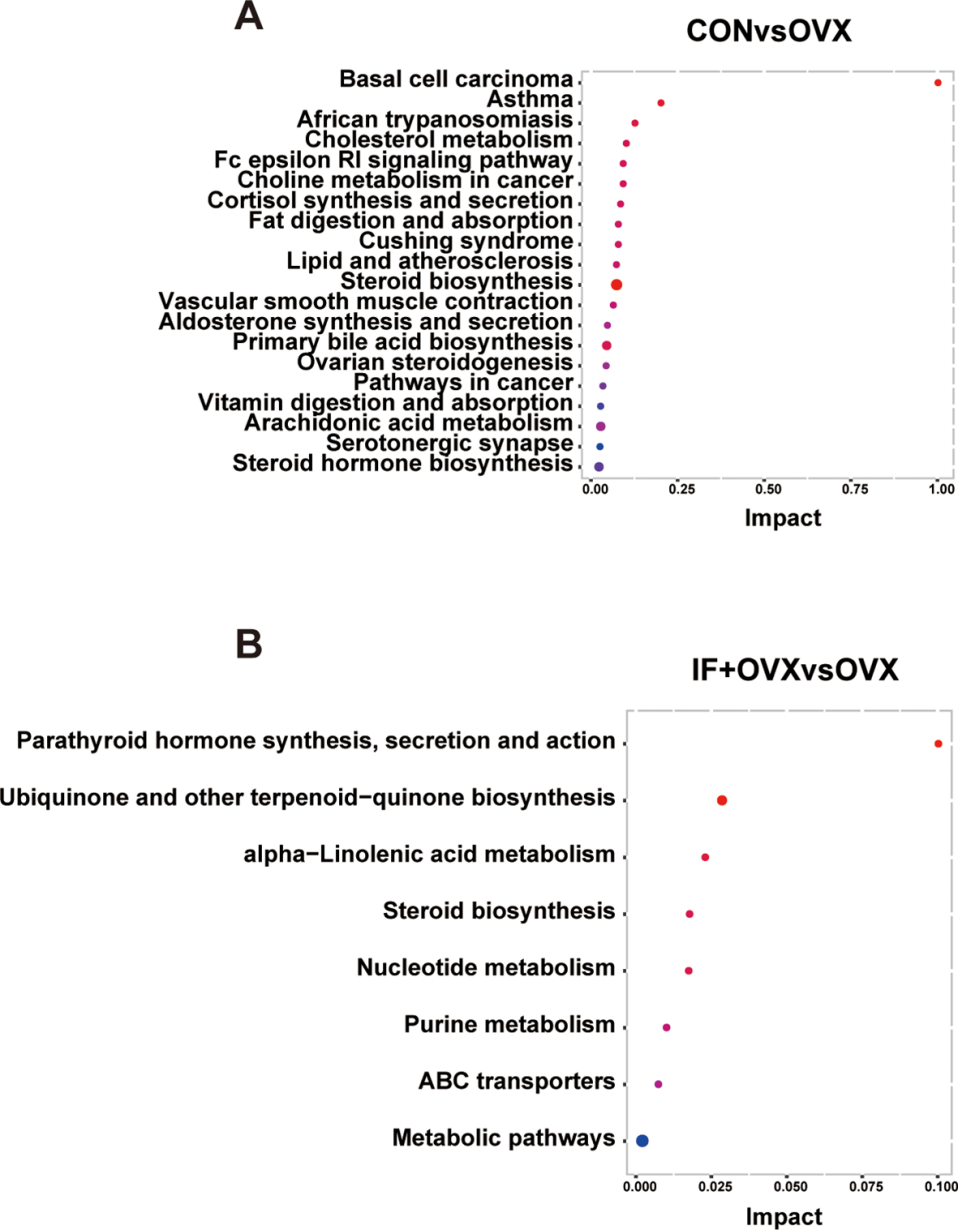
**(A)** KEGG pathway enrichment analysis annotation of differential fecal metabolites between the CON and OVX groups. **(B)** KEGG pathway enrichment analysis annotation of differential fecal metabolites between the OVX and IF+OVX groups. Data are presented as mean ± SEM. n = 7.

To explore the variations in fecal metabolites among the groups, KEGG pathway enrichment analysis was performed (**Figures 9A, B**). The results highlighted that the metabolic pathways influenced by oral *Isaria felina* primarily involved lipid and nucleotide metabolism. These pathways included alpha-linolenic acid metabolism, steroid biosynthesis, nucleotide metabolism, and purine metabolism.

### 3.5 *Isaria felina* regulates Th17/Treg immune imbalance induced by postmenopausal osteoporosis

**Figure 10:**
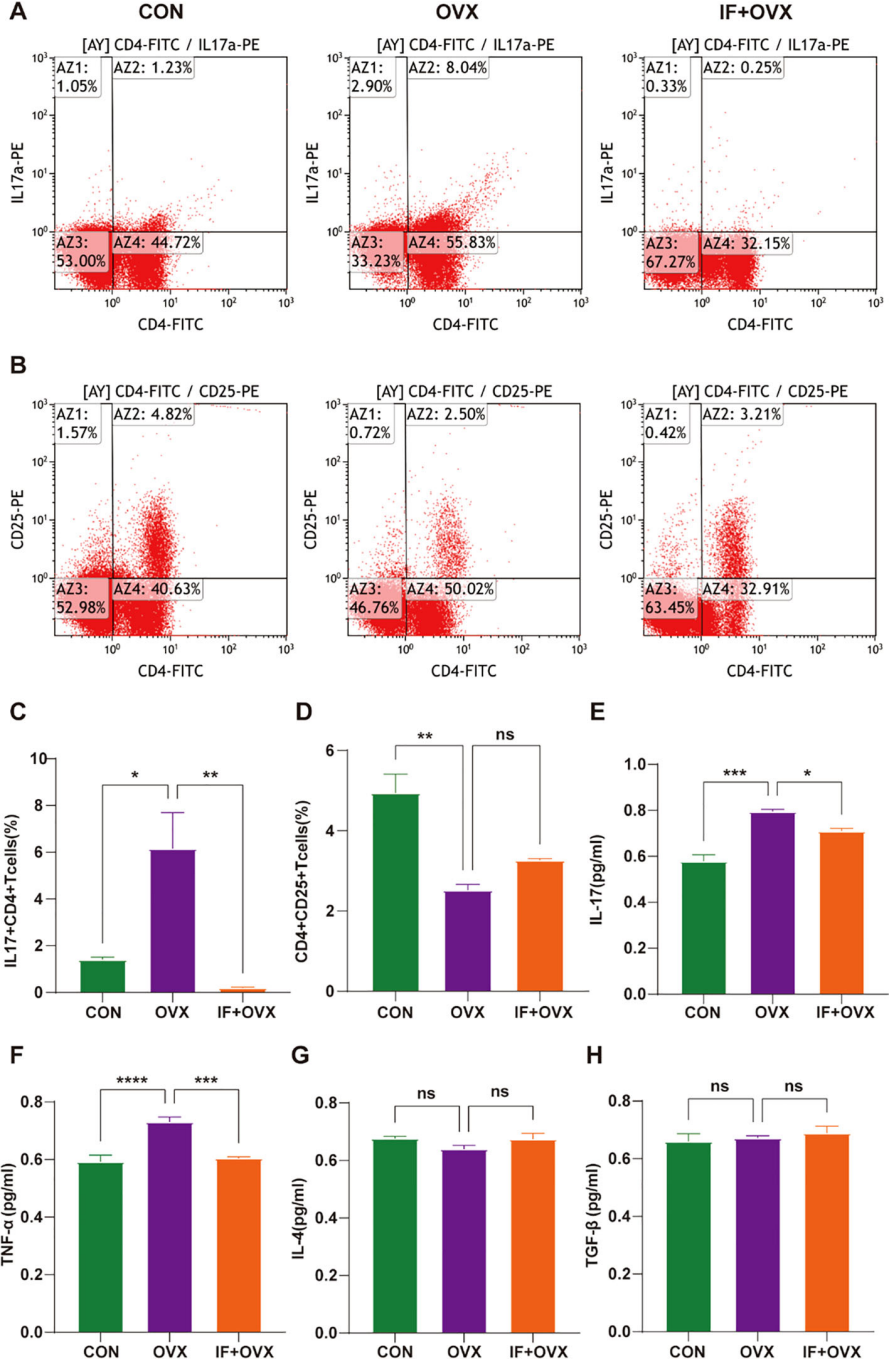
**(A)** Flow cytometry analysis of IL-17+CD4+ T cells in the peripheral blood of each group of rats. **(B)** Flow cytometry analysis of CD4+CD25+ T cells in the peripheral blood of each group of rats. **(C)** Quantification of Th17 cells. **(D)** Quantification of Treg cells. **(E-H)** Expression levels of serum inflammatory cytokines IL-17, TNF-α, IL-4, and TGF-β. Data are presented as mean ± SEM. n = 7, *P < 0.05, **P < 0.01, ***P < 0.001, ****P < 0.0001, ns represents no significance.

Peripheral blood was collected from rats via the abdominal aorta for flow cytometry analysis following oral *Isaria felina* treatment. The findings revealed a significant increase in interleukin-17-positive (IL-17+)+CD4+ T cells (Th17) and a notable decrease in CD4+CD25+ T cells (Treg) in the OVX group compared to the CON group. Subsequent treatment with oral *Isaria felina* significantly reduced Th17 cells and elevated Treg cells in the IF+OVX group compared to the OVX group, although the increase in Treg cells was not statistically significant (**Figures 10A–D**). Furthermore, ELISA assessed cytokines associated with Th17 and Treg cell secretion, including IL-17 and TNF-α for Th17 cells, and IL-4 and TGF-β for Treg cells. The results indicated that, relative to the OVX group, levels of IL-17 and TNF-α significantly decreased, while IL-4 and TGF-β levels increased in the IF+OVX group post-treatment, although the increases were not statistically significant (**Figures 10E–H**). These findings suggest that oral *Isaria felina* has the potential to modulate and rectify the Th17/Treg immune imbalance in OVX rats.

## 4 Discussion

Postmenopausal osteoporosis, the most prevalent form of primary osteoporosis, arises primarily from estrogen deficiency, leading to reduced bone mass and disrupted bone microstructure, thereby increasing fracture susceptibility. This systemic metabolic disorder poses a significant threat to women’s health worldwide (29, 30). Current clinical interventions for osteoporosis mainly include bisphosphonates, calcitonin, and estrogen therapies. Despite their efficacy, long-term use of these treatments is limited by side effects and adverse reactions. The application of traditional Chinese medicine (TCM) in osteoporosis management is gaining increasing attention from researchers and institutions globally (31). Consequently, identifying potential TCM targets for osteoporosis treatment is becoming increasingly important. This study aims to observe the therapeutic effects of *Isaria felina* on postmenopausal osteoporosis and explore its underlying mechanisms.

Imaging, histological, and mechanical assessments revealed significant improvements in bone loss in postmenopausal osteoporosis rats following oral administration of *Isaria felina*. Additionally, *Isaria felina* effectively managed obesity induced by postmenopausal osteoporosis. Studies have established a causal link between obesity and osteoporosis, where obesity adversely affects bone microstructure and may act as a risk factor for osteoporosis (32–34). Obesity prompts the differentiation of bone marrow mesenchymal stem cells (BMSCs) into adipocytes, increasing bone marrow fat levels and decreasing osteoblasts. Excessive bone marrow fat can replace bone cells with adipocytes, altering the bone microenvironment and microstructure and ultimately reducing bone density (35). Furthermore, excess adipose tissue releases numerous inflammatory factors, most of which activate the receptor activator of nuclear factor kappa-B (RANK) pathway, triggering osteoclast activity and causing bone loss (36). Histological examination of femurs from postmenopausal osteoporosis rats showed notable lipid droplet accumulation in the bone marrow cavity. However, treatment with *Isaria felina* reduced the number of lipid droplets in the bone marrow cavity. The effective management of obesity by *Isaria felina* indirectly highlights its role in treating osteoporosis. Nevertheless, further experiments are needed to validate the causal relationship between these observations.

Gut microbiota significantly influences the development and progression of osteoporosis (37–39), with studies demonstrating notable changes in gut microbial diversity in both animal models and human cases (3, 18, 39–42). Previous research confirmed alterations in gut microbiota associated with osteoporosis, identifying distinct changes for various types. In this study, 16S ribosomal DNA (16S rDNA) microbial detection revealed that *Isaria felina* treatment reversed gut microbiota dysbiosis in postmenopausal osteoporotic rats. Reversed bacterial genera included *Bacteroides, Tuzzerella, Ruminococcus_torques_group, Barnesiella, Parabacteroides, Helicobacter, Alloprevotella*, and *Frisingicoccus*. For instance, the genus *Ruminococcus_torques_group*, identified in previous experiments, is crucial for metabolism. Huang et al. found an increased relative abundance of *Ruminococcus* in osteoporosis groups (43), while Palmas et al. reported similar findings in obese patients (44). *Ruminococcus*, associated with active inflammatory bowel disease, stimulates immune system cells, such as tumor necrosis factor-alpha (TNF-α), which mediates the differentiation of osteoclasts and osteoblasts, contributing to postmenopausal osteoporosis (45). Thus, gut microbiota like *Ruminococcus* may play a key role in the therapeutic effects of *Isaria felina* on postmenopausal osteoporosis.

Metabolomics, emerging after genomics, transcriptomics, and proteomics, aims to quantitatively describe changes in metabolites within organisms (46). Positioned at the culmination of gene regulation and protein function networks, metabolomics reveals the ultimate state of biological processes, portraying ongoing events within the organism and elucidating upstream life activities’ intricate network structure (47). This study employed untargeted metabolomics to identify significant alterations in fecal metabolites in postmenopausal osteoporotic rats, with *Isaria felina* reversing changes primarily in nucleotide and lipid metabolism. Research links abnormalities in nucleotide, lipid, and amino acid metabolism to postmenopausal osteoporosis in mice (48). Lin et al. demonstrated that Bone Health Granule regulates bone metabolism by influencing nucleotide and amino acid metabolism and the immune system (49). Prior experiments also detected fecal metabolic disorders in various osteoporosis types, focusing on lipid and amino acid metabolism. Therefore, *Isaria felina* may influence bone metabolism by modulating metabolites associated with nucleotide and lipid metabolism.

Postmenopausal osteoporosis, a systemic skeletal condition, results from estrogen deficiency, leading to diminished bone mass, altered bone microarchitecture, and increased fracture susceptibility. Estrogen plays a crucial role in immune regulation, significantly impacting bone health through immune cells and their products (50, 51). T lymphocytes, originating from lymphoid stem cells in the bone marrow, undergo differentiation and maturation in the thymus, then disseminate to various immune organs and tissues to fulfill defensive roles. Regulatory T cells, a subtype of CD4+ T cells, regulate bone metabolism, with the equilibrium between T helper 17 (Th17) and Regulatory T (Treg) cells being pivotal in maintaining bone density (52). Estrogen deficiency disrupts gut barrier function, activates T cells, and increases Th17 cell populations in the intestinal mucosa, elevating Interleukin-17 (IL-17) production in the lamina propria (53). Th17 cells are crucial in estrogen deficiency-induced bone loss, as IL-17 promotes bone resorption, exacerbating bone deterioration. Clinical studies have demonstrated elevated IL-17 levels in the serum of patients with postmenopausal osteoporosis (54–56). Treg cells counterbalance Th17 cells and can transition between phenotypes, promoting anti-resorptive cytokines such as IL-10 and Transforming Growth Factor-beta (TGF-β), inhibiting osteoclast precursor apoptosis, and suppressing bone resorption (57). Modulating gut microbiota influences the balance between Th17 and Treg cells, favoring Treg cell expansion, inhibiting Th17 cell proliferation, reducing pro-inflammatory cytokine levels, and mitigating bone loss in postmenopausal osteoporosis (26). Studies have demonstrated that the interaction between the gut microbiota and the immune system is involved in the effects of estrogen deficiency on trabecular bone, with ovariectomy enhancing the migration of Th17 cells from the gut to the bone marrow (58). Estrogen deficiency leads to the expansion of gut microbiota-dependent Bone Marrow Th17 cells and TNF-α-producing T cells, which in turn upregulates osteoclast activity (59). Blocking the outflow of Th17 and TNF+ T cells from the gut or their inflow into the bone marrow prevents ovariectomy-induced bone loss, suggesting that blocking the migration of gut T cells could be a therapeutic strategy for treating postmenopausal bone loss (59). *Isaria felina* ameliorates bone loss in postmenopausal osteoporotic rats by modulating gut microbiota and immune regulation. Analysis of T lymphocytes and associated inflammatory cytokines in rat peripheral blood indicated that oral *Isaria felina* suppresses Th17 cell activity, leading to decreased expression of IL-17 and TNF-α, thereby attenuating osteoclast activation and mitigating bone loss in postmenopausal osteoporosis. The concept of the “gut microbiota-immune-bone axis” is introduced, suggesting further investigations to elucidate the regulatory interplay among these elements.

## 5 Conclusion

Oral administration of *Isaria felina* significantly improves bone loss and obesity in postmenopausal osteoporosis by targeting multiple mechanisms. This study demonstrates that *Isaria felina* treatment effectively reverses gut microbiota dysbiosis, modulates nucleotide and lipid metabolism, and restores the immune balance between Th17 and Treg cells. These findings highlight the intricate interplay of the gut microbiota-immune-bone axis in postmenopausal osteoporosis and suggest that *Isaria felina* exerts therapeutic effects by mitigating gut microbiota alterations and regulating immune responses. Future research should focus on elucidating the precise molecular pathways and validating these findings in clinical settings to support the use of *Isaria felina* as a potential therapeutic agent for postmenopausal osteoporosis.

## Data Availability

The datasets presented in this study can be found in online repositories. The names of the repository/repositories and accession number(s) can be found below: PRJNA1158011 (SRA) and OMIX009643 (NGDC).

## Supplementary material

Supplementary Figure 1
**(A)** Body weight monitoring of each group of rats. **(B)** Uterine weight of each group of rats after the treatment with oral Isaria felina. Data are presented as mean ± SEM. n = 7, ****P < 0.0001.
