# Supplementary figures and images for: Therapeutic effects of Isaria felina on postmenopausal osteoporosis: modulation of gut microbiota, metabolites, and immune responses
Source: Front Immunol. 2025 Apr 9;16:1508634. doi: 10.3389/fimmu.2025.1508634 (PMC12015163; doi:10.3389/fimmu.2025.1508634)

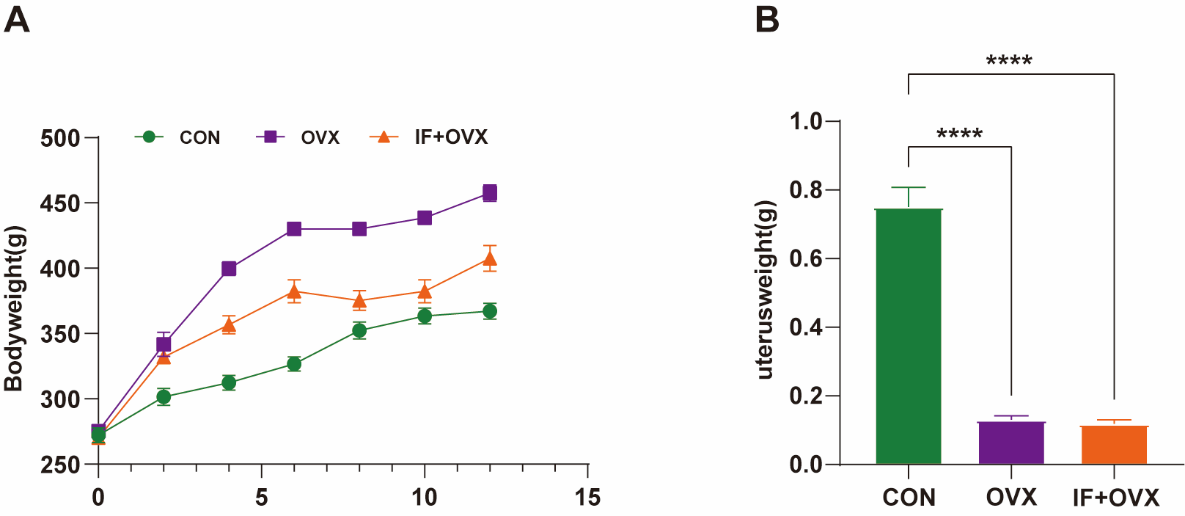

Supplement: Supplementary Figure 1 — (A) Body weight monitoring of each group of rats. (B) Uterine weight of each group of rats after the treatment with oral Isaria felina. Data are presented as mean ± SEM. n = 7, ****P < 0.0001. [file Image1.tif]
